# Explosive death induced by mean–field diffusion in identical oscillators

**DOI:** 10.1038/s41598-017-07926-x

**Published:** 2017-08-11

**Authors:** Umesh Kumar Verma, Amit Sharma, Neeraj Kumar Kamal, Jürgen Kurths, Manish Dev Shrimali

**Affiliations:** 1 Department of Physics, Central University of Rajasthan, Ajmer, 305 817 India; 2 The Institute of Mathematical Science, CIT Campus, Taramani, Chennai, 600 113 India; 3 Potsdam Institute for Climate Impact Research – Telegraphenberg A 31, 14473 Potsdam, Germany; 4 Department of Physics, Humboldt University - Berlin, 12489 Berlin, Germany

## Abstract

We report the occurrence of an explosive death transition for the first time in an ensemble of identical limit cycle and chaotic oscillators coupled via mean–field diffusion. In both systems, the variation of the normalized amplitude with the coupling strength exhibits an abrupt and irreversible transition to death state from an oscillatory state and this first order phase transition to death state is independent of the size of the system. This transition is quite general and has been found in all the coupled systems where in–phase oscillations co–exist with a coupling dependent homogeneous steady state. The backward transition point for this phase transition has been calculated using linear stability analysis which is in complete agreement with the numerics.

## Introduction

Synchronization^1, 2^ and suppression of oscillations^3–6^ are two most prominent emergent dynamics of the coupled oscillators. In synchronization, the collective dynamics of coupled system changes from incoherence to coherence. Although, the master stability function formalism^7, 8^ suggests that synchronization is a second order phase transition and the order parameter, which distinguishes coherence from incoherence, varies smoothly with the coupling strength, a non–trivial explosive synchronization^9, 10^ has been recently found in networks of coupled oscillator. The nature of phase transitions depends on the network topology and the characteristic dynamics of the oscillators in the network of dynamical systems. In the explosive transition, the order parameter exhibits an extremely abrupt jump at the transition point and shows a hysteresis behavior with distinct forward and backward transitions^10^. In the network of oscillators, adding even one new coupling, a connection between two oscillators or enhancing the coupling strength, can lead to the emergence of synchronized states^11, 12^.

Suppression of oscillations is another collective behavior of coupled oscillators and comes under two classes, namely, amplitude death (AD)^3, 4^ and oscillation death (OD)^5, 6^, depending on their origin. AD refers to a state, where all oscillators arrive at a common stable steady state, which is also a steady state of uncoupled oscillators, due to coupling. However, in case of OD coupled oscillators populate different coupling dependent steady states, which are created due to a coupling. This coupling seems to break the inherent symmetry of the oscillator and thus give rise to stable inhomogeneous steady states (IHSS) along with coupling dependent homogeneous steady states (HSS)^5, 6^. Since, all the oscillators populate the same steady states in HSS, this state is also termed as non–trivial amplitude death (NTAD) in the literature. AD is a widely studied topic due to its application in systems where suppression of unwanted oscillations is necessary, e.g.– in laser and mechanical engineering systems^13, 14^, etc., whereas OD has significant applications in biological systems, e.g.– cell differentiation etc.^15, 16^. Recently, there has been a lot of interest in understanding the transition from AD to OD in coupled oscillators and it has been found that a Hopf bifurcation in the coupled system stabilizes the trivial fixed point first, which on larger coupling gives birth to coupling dependent steady states known as HSS and IHSS through a pitchfork bifurcation^5, 6, 17^. Thus, the transition to death state from oscillatory state with coupling seems to be a second order phase transition.

However, an abrupt transition from an oscillatory state to a death one in coupled oscillators was also found^18^ where it was demonstrated that, apart from the Kuramoto model, an explosive transition might occur in a dynamical system involving amplitudes. This finding has extended the view of explosive transitions to amplitude models. It is well known that for the occurrence of a first order phase transition, two phases must co–exist over a parameter range. In many systems, e.g. in chemistry^19^ and physics^20^, the coexistence of stable oscillations and death has been demonstrated. Also, this situation has been found in various models of coupled limit–cycle and chaotic oscillators^21, 22^. Hence, it will be interesting to know the occurrence and mechanism behind any explosive transition from oscillatory to death state in a coupled system of identical oscillators. In this letter, we report the first evidence of a first–order phase transition with hysteresis in ensemble of identical oscillators. To study this phase transition, we consider an ensemble of limit–cycle and chaotic oscillators coupled via mean–field diffusion^23–28^.

## Results

To describe this explosive transition, we first consider *N* identical Van der Pol (VdP) oscillators coupled via a mean–field diffusion. The dynamics of this coupled system can be written as,

1 $$\begin{array}{rcl}{\dot{x}}_{i} & = & {y}_{i}+k(Q\bar{x}-{x}_{i}),\\ {\dot{y}}_{i} & = & b\mathrm{(1}-{x}_{i}^{2}){y}_{i}-{x}_{i},\end{array}$$

where, $i\,(=1,2,\ldots ,N)$ is the index of the oscillators. $b( > \mathrm{0)}$ indicates the non–linearity and strength of damping of a VdP oscillator. $\bar{x}=\frac{1}{N}{\sum }_{i\mathrm{=1}}^{N}{x}_{i}$ is the mean field of the state variable ‘$x$’. The parameter *Q*, with 0 ≤ *Q* <1 , is the intensity of the mean field and *k* is the strength of coupling.

To study the variation of the collective behaviour of systems with the coupling strength, *k*, we define an order parameter in term of average amplitude. The amplitude of each oscillator is numerically calculated by using the difference between the global maximum and minimum values of the time series of oscillator over a sufficiently long interval at a particular coupling strength *k*, defined as $a(k)=({\sum }_{i\mathrm{=1}}^{N}{\langle {x}_{i,max}\rangle }_{t}-{\langle {x}_{i,min}\rangle }_{t})/N$. The order parameter, *A*(*k*), which is the normalized average amplitude^23, 29^ is now defined as,

2 $$A(k)=\frac{a(k)}{a\mathrm{(0)}}\mathrm{.}$$

Thus, *A*(*k*), measures the average amplitude of all the oscillators in the coupled systems and for an oscillatory state the value of *A*(*k*) > 0, while for a death state *A*(*k*) = 0. We can also define another order parameter *E*(*k*)^29, 30^, which is the normalized mean incoherent energy of the system and is given by,

3 $$E(k)=\frac{\langle \sum _{j\mathrm{=1}}^{N}[{({{\bf{X}}}_{j}(k)-{{\bf{X}}}^{\star })}^{2}]\rangle }{\langle \sum _{j\mathrm{=1}}^{N}[{({{\bf{X}}}_{j}\mathrm{(0)}-{{\bf{X}}}^{\star })}^{2}]\rangle }$$

where **X**_*j*_(*k*) and **X**_*j*_(0) represent the state variables of the *j*-th oscillator at the coupling strength *k* and 0 respectively, **X** ^*^ represents the fixed point of the coupled system, and $\langle \mathrm{.}\rangle$ denotes the average over time. Clearly, the parameter *E*(*k*) has non–zero value when the system is in the active state.

The route to oscillation suppression can be numerically analyzed by computing the value of *A*(*k*) and *E*(*k*) adiabatically, both in forward and backward directions. Here, the dynamical equations are solved numerically for a random initial condition at some initial value *k* = *k*_*o*_ and *A*(*k*) and *E*(*k*) are calculated in the stationary regime. Then, we increase the coupling by a small value $\delta k$ and, using the outcome of the last run as the initial condition, calculate the new values $A({k}_{o}+\delta k)$ and $E({k}_{o}+\delta k)$. We repeat these steps until a maximal value *k*_*max*_ is reached. In the same way, the backward continuation is done by decreasing the coupling by steps of size $\delta k$ from the maximal value of *k*_*max*_. In all of the results presented here, we used $\delta k=0.02$, but our conclusions do not depend on this value of the increment.

**Figure 1:**
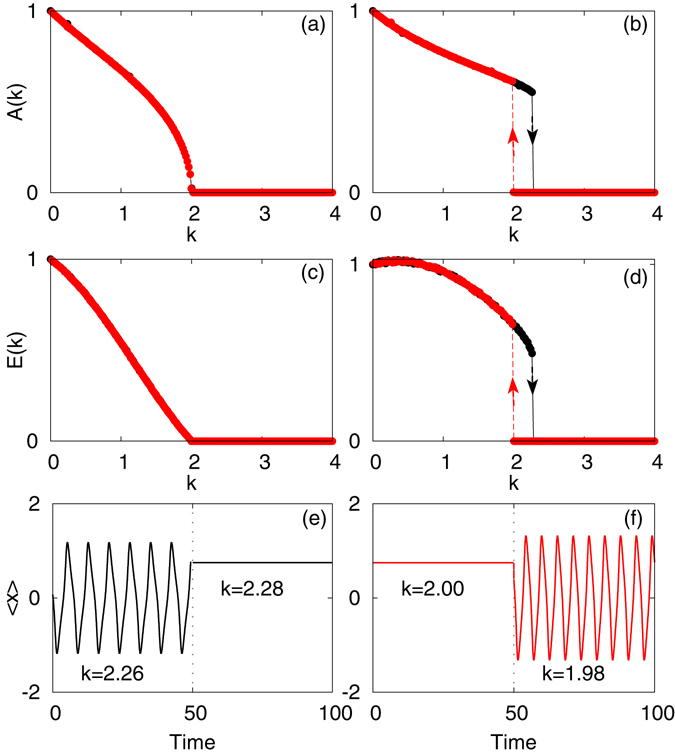
The transition from an oscillatory state to a quenched oscillation state of the coupled VdP oscillators is shown. Under the variation of the coupling strength, *k* both *forward* (black) and *backward* (red) continuations of the normalized amplitude, *A*(*k*), and normalized mean incoherent energy, *E*(*k*), are shown in (**a**) and (**c**) for *b* = 1.0 and (**b**) and (**d**) for *b* = 2.0. Time–series of the mean–field near the transition point for (**e**) forward and (**f**) backward continuations of Eq. (1) for *b* = 2.0. The other parameters are *Q* = 0.5 and *N* = 100.

In Fig. 1, the variation of *A*(*k*) and *E*(*k*), calculated by the procedure described above, is shown with *k*. Here, Fig. 1(a) and (c) exhibit a typical second order transition from oscillatory to death state and we find that the forward and backward transition point for *b* = 1 and *Q* = 0.5 are the same. However, in Fig. 1(b) and (d), which is shown for the parameter values *b* = 2 and *Q* = 0.5, we observe a sudden jump in the order parameters to *A*(*k*) = 0 & *E*(*k*) = 0 respectively in forward continuation which indicates the suppression of oscillations in the whole system. Similarly, the backward continuation also shows a sharp transition from *A*(*k*) = 0 & *E*(*k*) = 0 to a finite value which indicates oscillations in the system. These two transition points are at different value of *k*, and thus, a hysteresis area is observed which is reminiscent of a first order phase transition. The time dependent behavior of the mean–field, $< x > =\frac{1}{N}{\sum }_{j\mathrm{=1}}^{N}{x}_{j}$, is also shown in Fig. 1(e) and (f) for both forward and backward continuation respectively and for representative values of the coupling strength. It clearly indicates the abrupt transition of the amplitude as the strength of the coupling is changed.

**Figure 2:**
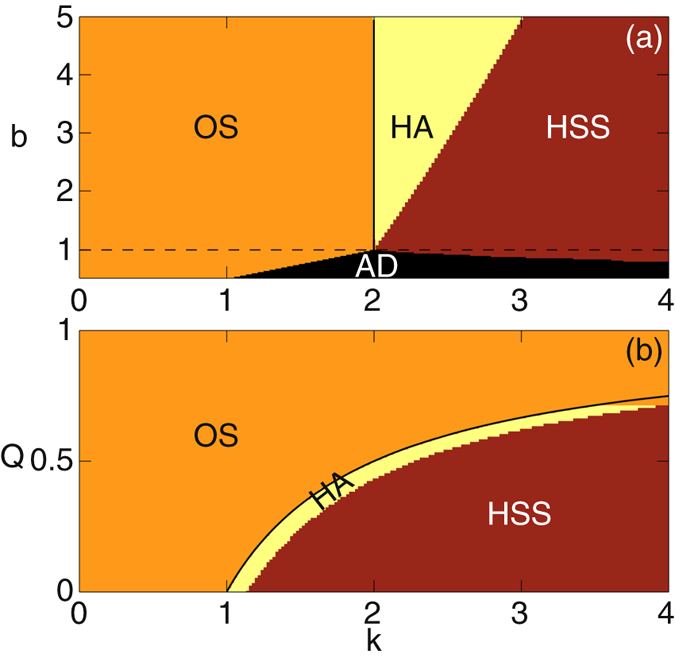
Different dynamical regions of the system described by Eq. (1) are plotted in the parameter planes. These dynamical regimes are observed over the range of value in (**a**) (*b* − *k*) parameter space at *Q* = 0.5, and in (**b**) (*Q* − *k*) parameter space at *b* = 2. The size of networks is *N* = 100. For details see the text.

The dynamical states of the coupled VdP oscillators obtained by both forward and backward continuations of *k* in the parameter space, are shown in Fig. 2. In this figure, OS, AD, HSS and HA represents oscillatory state, amplitude death, homogeneous steady state and hysteresis area respectively. Interestingly we uncover that the first order transition to death (HSS) occurs only when *b* > 1, while the second order transition occurs for *b* ≤ 1. In Fig. 2(a), the horizontal dashed line separates these two regions. We have found that, in the case of first order transition, the system is stabilized at the coupling dependent HSS, while in the case of second order transition, the system is stabilized at the origin. Also, the increase of *b* leads to an increase in HA in the parameter space (Fig. 2(a)).

The backward transition point for this explosive transition of this system can be computed by the stability analysis of HSS. The HSS for this system are, $({x}_{i}^{\ast }={x}^{\ast },{y}_{i}^{\ast }={y}^{\ast },\forall i=1,\ldots ,N)$, where ${x}^{\ast }=\sqrt{1-\frac{1}{bk\mathrm{(1}-Q)}}$ and ${y}^{\ast }=\sqrt{{k}^{2}{\mathrm{(1}-Q)}^{2}-\frac{k\mathrm{(1}-Q)}{b}}$. For this point, the 2*N* × 2*N* Jacobian matrices can be written in the form of a block circulant matrix^31^ **J** = circ (*A*, *B*, *B*…, *B*), where,

4 $$A=(\begin{array}{ll}\frac{kQ}{N}-k & 1\\ -2b{x}^{\ast }{y}^{\ast }-1 & b-b{x}^{\mathrm{\ast 2}}\end{array}),{\rm{and}}\,B=(\begin{array}{ll}\frac{kQ}{N} & 0\\ 0 & 0\end{array})\mathrm{.}$$

Thus, 2(*N* − 1) eigenvalues of the matrix **J** will be 2(*N* − 1) times degenerated and are equal to the eigenvalue of the matrix (*A* − *B*). The rest of the two eigenvalues will be equal to the eigenvalues of the matrix $A+(N-\mathrm{1)}B$ which decide the stability of the HSS. The characteristic equation corresponding to the matrix $A+(N-\mathrm{1)}B$ is,

5 $${\lambda }^{2}+{d}_{1}\lambda +{d}_{0}=\mathrm{0,}$$

where ${d}_{1}=\frac{{k}^{2}{\mathrm{(1}-Q)}^{2}-1}{k\mathrm{(1}-Q)}$ and ${d}_{0}=2bk\mathrm{(1}-Q)-2$. It gives the Hopf bifurcation point through which the HSS solutions are stabilized, which is,

6 $$k=\frac{1}{1-Q}\mathrm{.}$$

The value of *k* as a function of *Q* is shown by the solid black line in both Fig. 2(a) and (b). These analytical curves clearly separate the oscillatory region from the hysteresis area which are obtained numerically. Thus, for a particular value of the mean–field density, *Q*, the critical value of the coupling at which the backward transition occurs is given by Eq. (6). The coexistence of two different types of solutions, namely, oscillatory and HSS solutions, at the point of forward transition may be attributed to some kind of imperfect bifurcation due to which the oscillatory solutions lose their stability.

From this result, we can explain the phenomenology of this explosive transition. Here, an increase in *k* leads to synchronization in the coupled system. Due to the adiabatic evolution and co–existence of stable oscillatory solutions along with stable HSS solutions, the oscillatory solution persists for larger *k* in forward continuation. Once these oscillatory solutions loose their stability, the whole system go to stable HSS and there is an abrupt decay of *A*(*k*) and *E*(*k*). Similarly, during backward continuation, the system at HSS goes to stable oscillatory solutions once HSS loses its stability and there is an abrupt rise of the values of order parameters, *A*(*k*) & *E*(*k*), as can be seen in Fig. 1(b) and (d).

To generalize this mechanism, we now consider *N* chaotic Lorenz oscillators interacting via mean–field diffusion and whose dynamics is given by,

7 $$\begin{array}{rcl}{\dot{x}}_{i} & = & \sigma ({y}_{i}-{x}_{i})+k(Q\bar{x}-{x}_{i}),\\ {\dot{y}}_{i} & = & (r-{z}_{i}){x}_{i}-{y}_{i},\\ {\dot{z}}_{i} & = & {x}_{i}{y}_{i}-b{z}_{i},\end{array}$$

where, $i\,(=1,2,\ldots ,N)$ is the oscillator index. $\sigma =10,r=28$ and $b=\frac{8}{3}$ are the parameters of a chaotic Lorenz oscillator. *k* and *Q* are the strength of coupling and intensity of the mean field, $\bar{x}$, respectively.

**Figure 3:**
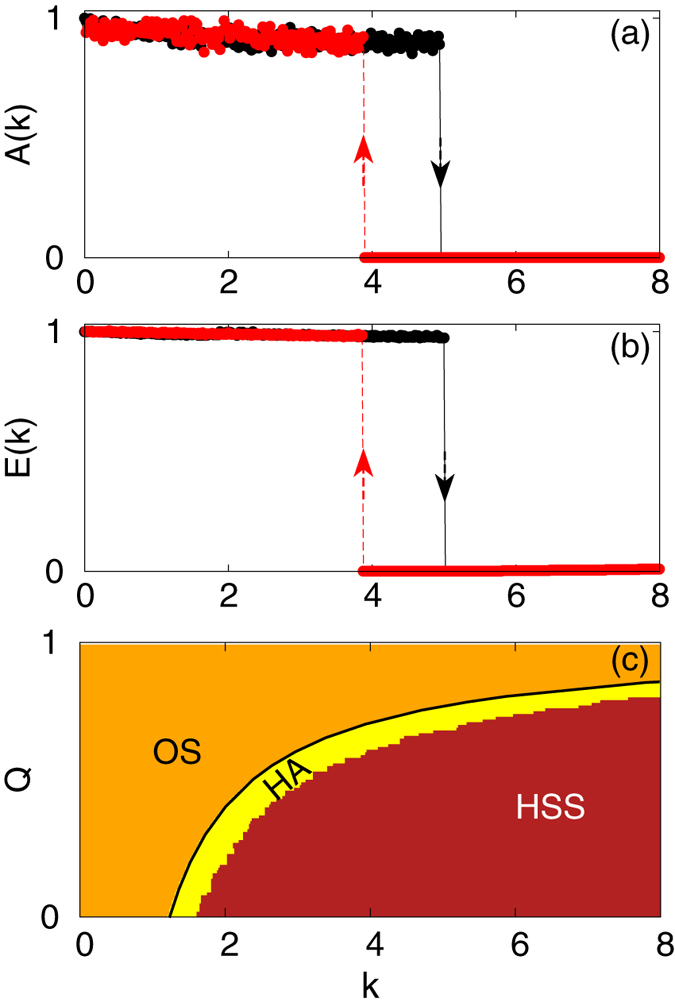
Forward (black) and backward (red) continuation diagram of (**a**) the normalized amplitude, *A*(*k*), and (**b**) normalized mean incoherent energy, *E*(*k*), under the variation of the coupling strength, *k*, for *Q* = 0.7 and (**c**) different dynamical regions in the parameter space of (*k* − *Q*), of *N* = 100 coupled Lorenz oscillators. For details see the text.

Figure 3(a) again exhibit an abrupt transition of *A*(*k*) in both forward and backward continuation indicating a first order transition to a quenched oscillation state from an oscillatory state and vice–versa. This statement is also well corroborated by Fig. 3(b) which shows the variation of another order parameter *E*(*k*) with *k*. The different collective behavior of this system over the variation of the parameters *k* and *Q* is shown in Fig. 3(c), using both forward and backward continuation. Here, we find three regions, namely, OS, HSS, and HA, where both oscillatory and steady state solutions co–exist. The backward transition point can also be calculated from the stability of HSS solutions which in this case are, $({x}_{i}^{\ast }={x}^{\ast },{y}_{i}^{\ast }={y}^{\ast },{z}_{i}^{\ast }={z}^{\ast },\forall \quad i=1,\ldots ,N)$, where, ${x}^{\ast }=\pm \sqrt{\frac{b\sigma (r-\mathrm{1)}-bk\mathrm{(1}-Q)}{\sigma +k\mathrm{(1}-Q)}}$, ${y}^{\ast }=\frac{\{\sigma +k\mathrm{(1}-Q)\}{x}^{\ast }}{\sigma }$ and ${z}^{\ast }=\frac{{x}^{\ast }{y}^{\ast }}{b}$. The Jacobian matrices corresponding to this point is a 3*N* × 3*N* matrix which can also be written in the form of a block circulant matrix^31^ **J** = circ (*A*, *B*, *B*…, *B*), where,

8 $$A=(\begin{array}{lll}\frac{kQ}{N}-k-\sigma  & \sigma  & 0\\ r-{z}^{\ast } & -1 & -{x}^{\ast }\\ {y}^{\ast } & {x}^{\ast } & -b\end{array}),\,{\rm{and}}\,B=(\begin{array}{lll}\frac{kQ}{N} & 0 & 0\\ 0 & 0 & 0\\ 0 & 0 & 0\end{array}).$$

The 3(*N* − 1) eigenvalues of **J** will be (*N* − 1) times degenerated and are equal to the eigenvalue of the matrix (*A* − B). The rest of the three eigenvalues will be equal to the eigenvalues of the matrix $A+(N-\mathrm{1)}B$ which give the stability of the HSS solutions. The characteristic equation corresponding to the matrix $A+(N-\mathrm{1)}B$ is,

9 $${\lambda }^{3}+{d}_{2}{\lambda }^{2}+{d}_{1}\lambda +{d}_{0}=\mathrm{0,}$$

where, ${d}_{2}=u+\sigma +k\mathrm{(1}-Q),{d}_{1}=b+\sigma u+ku\mathrm{(1}-Q)$ $+\,{x}^{\mathrm{\ast 2}}-v,{d}_{0}=\sigma {x}^{\ast }{y}^{\ast }+(\sigma +k-kQ)({x}^{\mathrm{\ast 2}}+b)$ $-bv,u=1+b$ and $v=\sigma (r-{z}^{\ast })$. From Eq. (9), the condition for stabilization of HSS is obtained and it is given by,

10 $$k=\frac{{u}^{2}+2u\sigma -v-\sqrt{m}}{2u(Q-\mathrm{1)}},$$

where, $m={b}^{4}-4{u}^{2}{x}^{\mathrm{\ast 2}}+4u\sigma {x}^{\ast }{y}^{\ast }+{\mathrm{(1}+v)}^{2}-2{b}^{2}\mathrm{(1}+v)$. This value of *k* as a function of *Q* is plotted in Fig. 3(c) by the solid black line and is the locus of the backward transition points of this first order transition.

**Figure 4:**
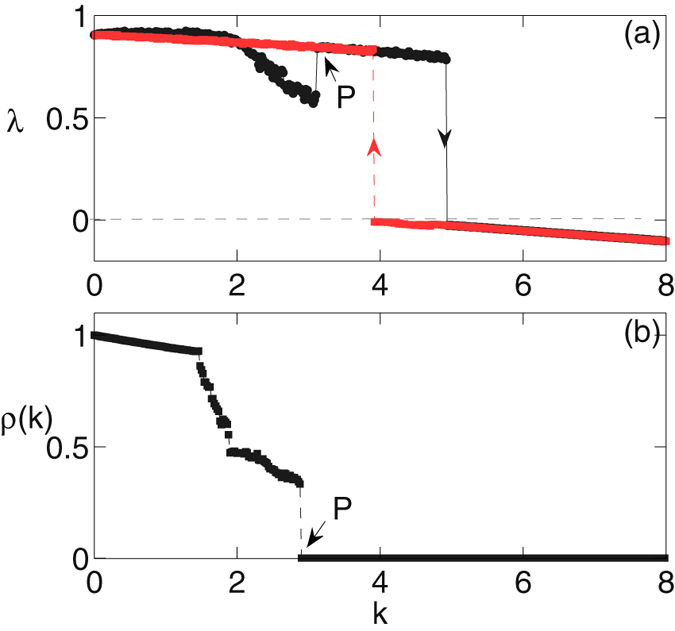
(**a**) The largest Lyapunov exponent, λ for both *forward* (black) and *backward* (red) continuations and (**b**) the normalized synchronization error, ρ(*k*) for *forward* continuation of *N* coupled Lorenz oscillators given by Eq. (7) as a function of the coupling strength, *k*. The marked point P denotes the onset of the synchronization regime. Other parameters are *N* = 100, and *Q* = 0.7.

Thus, the mechanism leading to this abrupt transition is similar to the mechanism discussed for the case of coupled VdP oscillators. To corroborate more, we calculate the variation of the largest Lyapunov exponent of this system on *k* which is shown in Fig. 4(a). Here, the largest Lyapunov exponent drastically changes its value from positive to negative at the point of transition. Thus, the coupled system is in a chaotic state before the transition and after that it settles to coupling dependent homogeneous steady state. The probability density of largest finite time Lyapunov exponent for this coupled system also indicate the transition from a chaotic state to a fixed point state at the point of transition. For more understanding of this phenomena, we also calculate the normalized synchronization error, $\rho (k)=\frac{S(k)}{S\mathrm{(0)}}$, as a function of *k*, where,

11 $$S(k)=\langle \sqrt{\frac{1}{3N}\sum _{i\mathrm{=1}}^{N}[{({x}_{i}-\bar{x})}^{2}+{({y}_{i}-\bar{y})}^{2}+{({z}_{i}-\bar{z})}^{2}]}\rangle \mathrm{.}$$

Here, $\mathrm{ < .. > }$ represents the long time average. Clearly, for complete synchronization $\rho (k)=0$. The plot of ρ(*k*) for various values of *k* is shown in Fig. 4(b). Here, both, the maximum Lyapunov exponent and the normalized synchronization error are calculated adiabatically. From Fig. 4(a) and (b), it is clear that all oscillators first synchronize among themselves at a critical value of coupling which is shown by the point ‘P’ and as the coupling strength increases further the oscillatory solutions loose their stability and the system goes to HSS.

## Discussion

To conclude, we have studied the suppression of oscillations from oscillatory state of an ensemble of limit–cycle and chaotic oscillators from phase transition point of view. We have shown for the first time that this transition can be of first order with hysteresis in systems of identical oscillators. The underlying mechanism of this explosive transition is also discussed which is qualitatively the same for limit–cycle and chaotic oscillators. Although the results presented here are for the case *N* = 100, we have found these independent of the number of oscillators in the system. Apart from explosive death, the bistability in terms of activity and quiescence in these systems can also be accounted for the observed phenomena of annihilation and single–pulse triggering in biological systems^32^. The ceasing of spontaneous activity in a system by applying a sub–threshold pulse is termed as annihilation and it has been observed in the eclosion rhythm of fruit flies, the circadian rhythm of bioluminescence in marine algae, the sinoatrial node in the heart and the Hodgkin–Huxley equations^32–35^. Also, injection of a supra–threshold current pulse in the system, restarts the activity from quiescence which is termed as single-pulse triggering and has been observed in the sinoatrial node in the heart and the Hodgkin–Huxley equations^33–35^. In all these biological systems, there must be a bistability of oscillatory and steady state solutions. Once the forcing in form of a signal is applied to them, the nature of the dynamics changes and the system shows activity from quiescence or vice–versa. We hope, our studies will not only provide the mechanism for these biological processes but also provide a better understanding of explosive transitions in coupled dynamical systems. In diluted networks, emergence of first order transition to synchronized states have been found due to adding a new connection in the coupled oscillators^12, 36–38^. Although, we have studied the transition from active to inactive state in globally coupled systems, it will be interesting to study the effect of adding or removing a link between the nodes in complex networks on these explosive transitions.
